# Migraine with extensive skin markings: a case report

**DOI:** 10.1186/s12245-018-0191-x

**Published:** 2018-06-07

**Authors:** Sabrina Berdouk, Sobia Khan

**Affiliations:** Al Qassimi Hospital, Sharjah, United Arab Emirates

## Abstract

**Background:**

Migraines are a commonly seen complaint in the emergency department, but migraines with extensive skin markings have rarely been reported and so the cause is still unknown.

**Case presentation:**

We report a 28-year-old Pakistani male complaining of recurrent migraines that are now associated with skin markings that appear on his forehead and last for a few days and then disappear on their own. A diagnosis of migraines with cutaneous markings was made. An MRI was done on the patient and showed normal results. The patient has only been given painkillers for the treatment of his migraines. No other treatment or interventions have been done.

**Conclusion:**

The cause of these markings is still unknown, and very little literature has been written on such cases. Those markings appear to be self-resolving as the migraines subside. More investigations and research have to be done in order to find the cause of such markings, establish the natural history, and enhance their management.

## Background

Migraines are amongst the most common complaints seen in the emergency department. In the USA, around 12% of people suffer from migraines [1].

Migraines usually are described as progressive onset unilateral headaches increasing in intensity.

The patient generally describes the pain as throbbing or pulsatile frequently associated with photophobia or phonophobia [1]. Most textbooks describe associated prodromes, postdrome, and aura symptoms such as visual changes, sensory changes, and sometimes depression, fatigue, and irritability [1]. Migraines with cutaneous symptoms are quite a rare phenomenon. This manifestation has only come to light in recent years, and the etiology is still not clearly determined. Furthermore, there is very little literature on such symptoms. There are only a handful of case reports about this topic referred to as the “Red dot syndrome” and the “Red ear syndrome.”

The purpose of our paper is to present the case of a 28-year-old Pakistani male with chronic migraines associated with extensive cutaneous markings.

## Case presentation

A 28-year-old Pakistani male presented to the emergency department (ED) complaining of a severe headache. The headache had started 7 days prior to presentation and was localized to the left side of the head with no radiation. The pain was pulsatile and throbbing in nature. The patient rated the pain 9/10 on the visual analog scale. His headache was associated with unilateral photophobia and denied any visual impairment. There were no other associated symptoms and no focal neurological abnormality. The patient had a long-standing history of migraines that started around 2 years ago, experiencing an episode once every 2 weeks. Usually, those episodes subsided with paracetamol and Ibuprofen; on the rare occasion, the patient has had to present to the ED for further management.

**Fig. 1:**
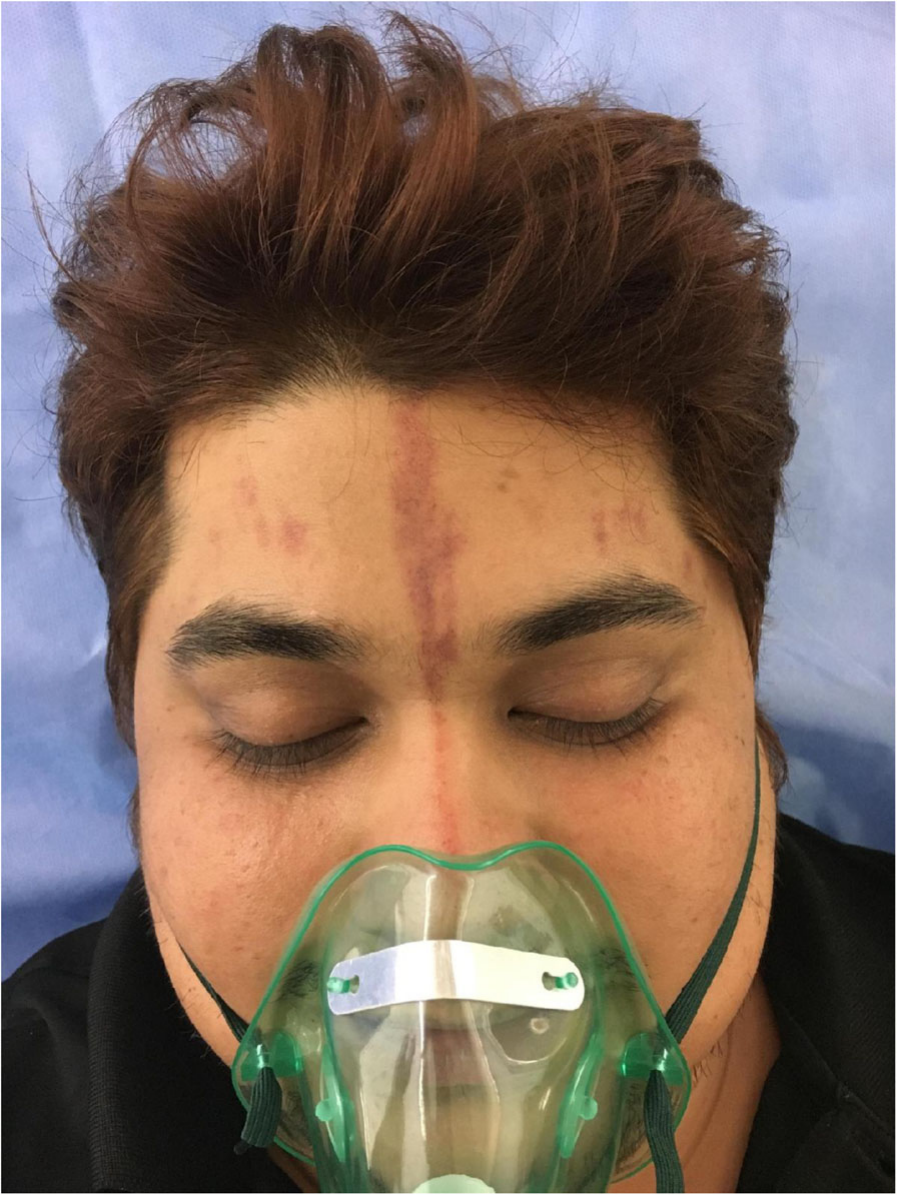
The cutaneous markings from his forehead down to his nose. This picture was taken in the ED department showing the cutaneous markings present on his forehead. The markings had appeared a few days after the onset of the headache and were getting worse in color and size

**Fig. 2:**
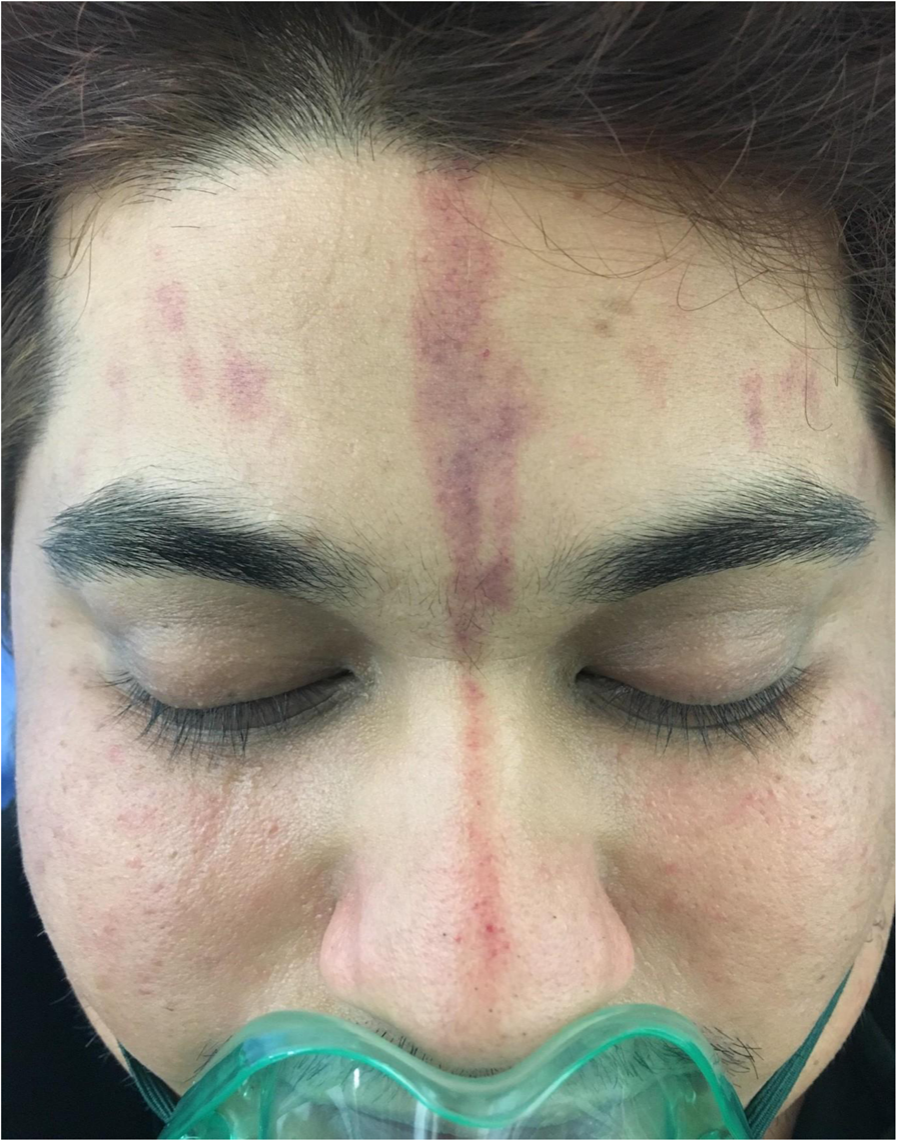
A closer look at the same markings. This is a closer look at the same markings; this picture was also taken in the ED at the same time as the previous picture. In this picture, you can see the color and size of the markings more clearly and can see that it is very prominent

**Fig. 3:**
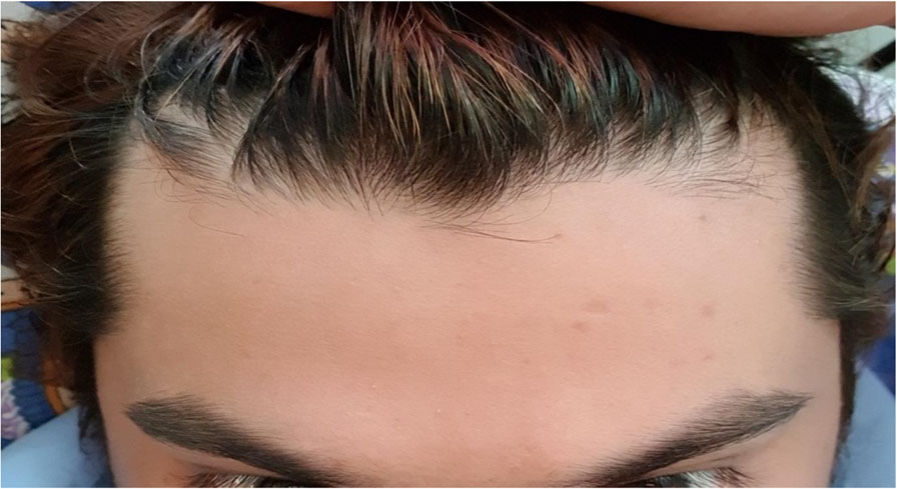
Patients forehead after the markings disappeared. This picture was taken by the patient a few days after he was discharged and the migraine had settled. As you can see, the markings had totally disappeared and his forehead was completely clear. This is what happens each time the markings appear, they completely disappear after the migraine has gone

Along with his migraine, our patient developed cutaneous markings (Figs. 1 and 2). There were multiple, 1–2-cm non-blanching linear erythematous lesions that appeared on his forehead and disappeared a few days after the headache subsided (Fig. 3). These markings, first appeared in 2014, then reappeared in April 2017 and had been reoccurring with each migraine attack until he visited our ED. The patient correlated the severity of the pain with the size and number of markings. He reported that as his pain increased, the marks would get more intense in color, they started off as red but then eventually would turn blue/black resembling a bruise.

The patient’s past medical history was only significant for dyslipidemia, for which he was on a statin. Of note, the patient has a positive family history for migraines, but denies any migraine-associated cutaneous manifestation in the family. On presentation to the emergency department, the patient vitals were as follows: temperature 36.8 °C, a pulse rate of 118 bpm, a respiratory rate of 18 bpm, and a blood pressure of 148/91 mmHg. On examination, he was alert, oriented to time and place. On further examination, his chest and lungs were clear and he had no focal neurological deficits. His pupils were reactive and his GCS was 15. His forehead showed multiple 1–2-cm erythematous markings that were purple in color and non-tender (as shown in pictures 1 and 2 below). We put the patient on an oxygen mask and gave him 20 mg of metoclopramide IM. The patient was then kept in a dark room to rest, and within 30 min, the patients pain decreased from a severity of 9/10 to a 4/10. Laboratory investigations were not sent in this visit as the patient felt significantly better and had had prior workup done, including an MRI, which were all unremarkable. Although the patient’s pain had significantly improved, the markings were the same. They remained the same size and shape upon discharge. The patient was advised to follow up with a neurologist for further management. When the patient was then later contacted, the markings had disappeared on their own a few days later (Fig. 3).

### Differential diagnosis

Our top differential diagnosis was migraines, as the patient’s history was classic of migraines. But we also thought about the possibility of a brain tumor, tension headaches, cluster headaches, and even possibly a vascular disorder. Acute headache causes were ruled out due to the patient’s history of frequent similar episodes of headaches. As the patient has been fully worked up, we ruled out most pathologies including a brain tumor as the MRI was reported normal.

### Clinical diagnosis

The clinical diagnosis was migraines with cutaneous manifestations.

## Discussion

Only a very minimal number of such cases have been reported, but none with such extensive cutaneous markings. One of the first cases was reported by Sethi et al. [2], a 40-year-old male with classic migraine symptoms presented with a red dot on his forehead extending along the V1 distribution of the trigeminal nerve. His workup was all normal [2]. Another case was reported by Sethi in 2015, a 32-year-old woman with episodic headaches with a similar red dot on her forehead accompanied by ecchymosis on her eyelid in the V1 distribution of the trigeminal nerve [3]. Monaghan [4] reported a 44-year-old-woman with periocular bruising 2 days after the onset of her headache. Although all these patients presented with a classical migraine history like our patient and each of them developed cutaneous manifestations, none of them have had such severe markings. Our patient’s markings, unlike Sethi’s, did not follow the V1 distribution and were much more prominent in size and number.

## Conclusions

Migraines with cutaneous manifestations are a very rare presentation and have only recently been discovered. A lot more research needs to be done including more testing to find out the etiology.

Genetic testing can possibly be undertaken to see whether it could be related to a gene mutation and hence further enhance the management of those patients. Furthermore, patients with this condition require additional follow-up to determine the natural history and progression of the skin manifestations that occur with those migraines.

## Consent

Written consent for publishing this case report including the images was taken from the patient.

## Abbreviations

ED: Emergency department
IM: Intramuscular
MRI: Magnetic resonance imaging
